# Preliminary Study of Scent Rolling in Captive Wolves (*Canis lupus* L. 1758) in Response to Olfactory Enrichment

**DOI:** 10.3390/biology13060422

**Published:** 2024-06-06

**Authors:** Nikolina Boić, Nikica Jukić, Alma Mikuška, Dora Bjedov, Mislav Kovačić, Tatjana Šalika-Todorović, Mirta Sudarić Bogojević

**Affiliations:** 1 Department of Biology, Josip Juraj Strossmayer University of Osijek, Cara Hadrijana 8a, 31000 Osijek, Croatia; 2 Public Institution Aquatika—Freshwater Aquarium Karlovac, Branka Čavlovića Čavleka 1a, 47000 Karlovac, Croatia; 3 UNIKOM d.o.o., Osijek Zoo, Sjevernodravska obala 1, 31000 Osijek, Croatia; 4 Centre for Ecology, Evolution and Environmental Changes (cE3c) & CHANGE—Global Change and Sustainability Institute, Faculdade de Ciências, Universidade de Lisboa, Campo Grande, 1749-016 Lisbon, Portugal; 5 Departamento de Biologia Animal, Faculdade de Ciências, Universidade de Lisboa, Campo Grande, 1749-016 Lisbon, Portugal

**Keywords:** wolves, scent rolling, odour, olfactory enrichment, behaviour

## Abstract

**Simple Summary:**

Scent rolling, a behaviour observed in large carnivores like wolves, involves lowering the chin and neck towards a scent, then rubbing the head, neck, shoulders, and back into it. Despite its prevalence, the exact reason for this behaviour remains unknown. In this study, captive wolves at Osijek Zoo responded differently to various odours presented during olfactory enrichment. In the first year, odours like curry, rosemary, and deer/mouflon and rat faeces garnered the highest interest in scented objects and scent-rolling behaviour. In the second year, llama faeces and deer/mouflon faeces elicited longer interest, while others, like guinea pig faeces and oregano, prompted less interest. During the second part of this study, it was observed that only females exhibited scent-rolling behaviour, indicating their higher level of engagement with the scents. Scent rolling did not occur in response to certain odours, suggesting the existence of selective preferences. Sheep’s wool prompted the longest scent-rolling sessions. Differences in behaviour between enrichment sessions were not significant; however, mornings generally elicited greater interest in odours. Despite various theories proposing alternative explanations for scent rolling in wolves, it seems to be triggered by novel odours.

**Abstract:**

Scent rolling, a behaviour observed in various large carnivores like wolves, entails the animal lowering its chin and neck towards a scent, followed by rubbing the head, neck, shoulders, and back into it. This behaviour is prevalent among wolves exposed to diverse scents, though its exact purpose remains uncertain. In this study, captive wolves at Osijek Zoo responded differently to odours during olfactory enrichment sessions. In the initial year of this study, the highest level of interest, evidenced by both the frequency of responses and scent-rolling behaviour, was noted when the wolves encountered odours such as curry and rosemary, along with deer/mouflon and rat faeces. While certain odours, such as llama faeces and deer/mouflon faeces, garnered longer durations of interest in the second year of study, others, like guinea pig faeces and oregano, elicited shorter responses. Female wolves demonstrated a higher level of engagement with scents compared with males, particularly through scent rolling behaviour, which was exclusively observed in females during the second year of this study. Interestingly, certain odours did not trigger scent rolling, suggesting selective preferences. On the other hand, sheep’s wool induced the longest duration of scent rolling, and a lack of significant differences in behaviour was observed between morning and afternoon sessions. Despite the existence of multiple hypotheses put forward to explain the causation of scent rolling in wolves, it seems to be elicited by unfamiliar odours.

## 1. Introduction

Early humans recognized the remarkable sense of smell in wolves and utilized it in hunting, fostering the development of human–wolf cooperation and the process of domestication [1]. This close relationship between humans and dogs, descendants of the domesticated subspecies *Canis lupus familiaris* Linnaeus, 1758, underscores the importance of understanding scent-related behaviours in canids (family Canidae), such as scent rolling, which is a specific behaviour observed in all large carnivores [2,3]. Wolves roll in a large number of different scents. This behaviour begins by sniffing the scent and lowering the head and neck towards it, which is followed by the rubbing of various body parts against the encountered scent. After the head and neck, wolves lower their shoulders and eventually their entire back, covering themselves with the encountered scent [4]. The lips, chin, neck, cheeks, shoulders, back of the head, chest, and back are the most common places to rub [3]. 

Wolves use scent marking as a means of communication, effectively establishing an “information centre” within their territory [5]. Through scent rolling, wolves deposit scent from glands situated in their neck and back regions onto various surfaces like rocks, trees, or the ground. These scent markings play a pivotal role in conveying crucial information concerning pack territory, social hierarchy, and reproductive status to neighbouring wolves [6]. While the precise motivation behind this behavior remains elusive, various theories offer potential explanations [4]. It is possible that wolves engage in this behavior either to mask their own scent or to leave their scent on an object [3,7]. Another hypothesis suggests that scent rolling may serve to reinforce social bonds within the pack and establish pack hierarchy, or to communicate some information to other pack members [8]. What is certain, however, is that this behaviour is triggered by the presence of a new and previously unidentified odour, including scents not typically encountered in the wolf’s environment, familiar scents with altered characteristics, and scents to which wolves may have a strong aversion or attraction [4]. 

Scent rolling has been described as an unconditional response to strong odours [9], often viewed in animals like canids and felids as a Fixed Action Pattern (FAP). FAPs are innate, stereotyped behaviours specific to a species, triggered by particular olfactory cues. They can be influenced by learning, experience, or environmental factors. FAPs are a subset of Modal Action Patterns (MAPs), which encompass a broader range of motor actions observed across species for various purposes such as grooming, marking territory, or other behaviours (e.g., abdomen rubbing in some animals). In essence, a Fixed Action Pattern (FAP) is a specific type of Modal Action Pattern (MAP) characterized by its stereotyped nature and innate triggering mechanism. These behaviours are discussed within the large framework of understanding the physiological and motivational aspects of animal behaviour [10].

The number of studies devoted to olfaction varies strongly between 38 species from the family Canidae [11]. The majority of studies on olfaction have primarily focused on domestic dogs [12,13], with the assumption that the findings obtained for dogs could be extrapolated to other members of the canid family, suggesting that responses to odours are likely to be comparable across species [14]. For example, in some studies devoted to dog olfaction, French lavender and rabbit scents were used [12]. Even explosives were used when the research was conducted with working dogs or scent hounds [15]. A lesser number of studies have explored olfaction in other canid species, particularly wolves. In such cases, dried herbs [16] or industrial odours like perfumes were used [4]. Because of its remarkable sensitivity, the sense of smell is a suitable target for enrichment in captivity [17] and therefore the application of different scents can provide a stimulating environment for animals [16]. 

Enriching the environment of captive animals reduces stereotypical behaviours such as pacing around enclosures [18,19]. Stereotypical behaviours are defined as behaviours that have no obvious purpose [20], are repetitive, and are hard to change [21]. Olfactory enrichment has been used to enrich the environment of dogs housed in shelters in order to encourage them to increase physical activity and also to reduce stress levels [17,21]. One of the goals of enrichment is to encourage individuals to not only explore the environment but also to exhibit a greater range of behaviours [16]. Furthermore, environmental enrichment can be used to encourage species-specific behaviour, especially in animals kept in zoos [22], as such behaviour may be absent due to feeding regimes that do not meet the specific needs of these animals [23]. The most common odours used in olfactory enrichment include spices, food scents, essential oils, the urine and faeces of other animal species, and numerous artificial fragrances [24]. 

The aim of this study was to assess what stimuli will encourage captive wolves to engage in a specific form of behaviour, i.e., scent rolling. Another goal was to determine which odours elicited the most intense and long-term response, and to check if certain odours elicited the same response in all individuals. We expected the odour of herbivore faeces to elicit the highest level of sustained interest among zoo wolves, since these animals were bred in captivity without direct exposure to herbivores and their excrements. We hypothesized that these odours would be most attractive to wolves due to their novelty, resembling the natural prey they encounter in the wild, thus sparking their curiosity and potentially triggering the strongest instinctual response and prompting scent-rolling behaviour. We expected to observe differences in responses to the same odour between the morning and afternoon sessions, anticipating that the wolves’ reactions might vary due to potential changes in their activity levels or environmental conditions throughout the day.

## 2. Materials and Methods

The first part of the study involved conducting environmental enrichment activities over four days in November and December 2020 at the Osijek Zoo in Croatia. The primary aim of this part of the study was to determine which specific odour prompted the observed behavioural patterns. Wolves (*Canis lupus* L. 1758) were located in an enclosure of 6000 m^2^, rich in shrubs, trees, and fallen trunks, which provided conditions as close as possible to those encountered in their natural environment. Numerous trees, a wooden platform, and a lair in the enclosure provided enough space for play, rest, and shelter. The enclosure was completely fenced with a wire fence and an electric shepherd. A five-member wolf pack consisted of a young breeding couple (2 years old) and their three pups (two males and one female). The alpha male belongs to the subspecies *Canis lupus lupus* L. 1758, while the others are of the species *Canis lupus* L. 1758 with unknown origin and subspecies. At that time, the cubs were 6 months old. Wolves were fed meat from verified sources every other day.

The enrichment was conducted in the morning hours to avoid a large number of visitors. Each of the four days of enrichment, two different odours were simultaneously presented to the wolves. First, curry and rosemary, then, vanilla and lemon aromas, next, deer/mouflon and rat faeces, and finally, llama and guinea pig faeces were simultaneously given to the wolves. Odours were placed at opposite ends of the enclosure, approximately 20 m apart; therefore, the reactions to each odour could be observed separately. The aromas of vanilla and lemon were dropped on the branches, which were later inserted into the enclosure. The spices were placed in five paper bags to make them easier to insert into the enclosure, while the faeces mixed with straw were stored in boxes and thus placed in the enclosure. Approximately 15 mL of aromas, 20 g of spices, and 100 g of excrement were used for the research. Faeces were obtained from the herbivores of the Osijek Zoo. The number of items was determined by the principle that each wolf has its own item and an additional one (a general rule accepted in enrichment workshops; Šalika-Todorović, *pers. comm.*), i.e., the number of animals in the enclosure plus one, meaning that twelve odour stimuli (six items of each odour) were presented simultaneously to the five wolves on each day. The behaviour was documented using a digital camera (Sony Cyber-Shot). Observation occurred for two hours following the introduction of the odour into the enclosure, with only the wolves’ responses to experimental odours being recorded. The observer maintained a distance of one meter from the enclosure fence while monitoring the behaviour of the tested wolves.

The second part of this study was carried out in October 2021 with some minor modifications. This year, only four wolves participated in the experiment since one younger wolf was sent to the zoo in Poland. Therefore, the pack now consisted of a wolf pair and their two offspring (a male and a female). The experiment was carried out for 10 days in a row, and the scents were presented to the wolves twice a day. During the initial day of the experiment, wolves were not exposed to any scents, and their baseline behaviour was observed for one hour in the morning and one hour in the afternoon. On the subsequent 8 days, the wolves were introduced with one scent in the morning (9:00–10:00) and again in the afternoon following feeding (16:00–17:00). The scents were similar to the previous experiment, with the exception that, this time, cinnamon was used instead of lemon aroma, oregano instead of rosemary, and sheep wool instead of rat faeces (Table 1). On the final day, no odours were given to observe the post-enrichment behaviour. Post-enrichment behaviour was also observed during two one-hour sessions, mirroring the procedure applied on days during which odours were presented to the wolves. Both baseline and post-enrichment behaviour included various engagements, such as walking, running, lying down, and interactions between individuals, during periods of activity and rest without any external stimuli, excluding those introduced through our conducted enrichment activities. However, analysing these behaviours was not the focus of this study.

An ethogram (Table 2) was compiled to guide the observation of individual behaviours. Both the frequency and duration of specific behaviour patterns were scored through direct observation and camera recording (photographing). This ethogram was compiled and adopted on the basis of one already used in a previous study by another author focusing on wolf olfaction [25]. The analysis centred on sensory (olfactory) enrichment as a behavioural category, encompassing the behavioural patterns outlined in Table 2 [25]. This study was carried out as part of the daily enrichment program in the Osijek Zoo, which is normally carried out by professional zoo employees. 

The data were statistically processed using R 3.5.0 [26] and the RStudio Team [27]. The Shapiro–Wilk test revealed that the data were not normally distributed, prompting the use of non-parametric tests for further analysis. The Kruskal–Wallis test was used to investigate if there were significant inter-individual differences in the frequencies and durations of the analysed behavioural categories of wolves, as well as the frequencies and durations of behaviours in response to the different odours presented to them. Differences were determined by pairwise comparisons of the mean ranks for each column, followed by Dunn’s post hoc test. The Wilcoxon signed-rank test was used to compare the response durations obtained during the morning and afternoon sessions for the same scent, indicating that we pooled all scents together for this analysis. The level of statistical significance was 0.05 (*p*-value). 

## 3. Results

**Figure 1:**
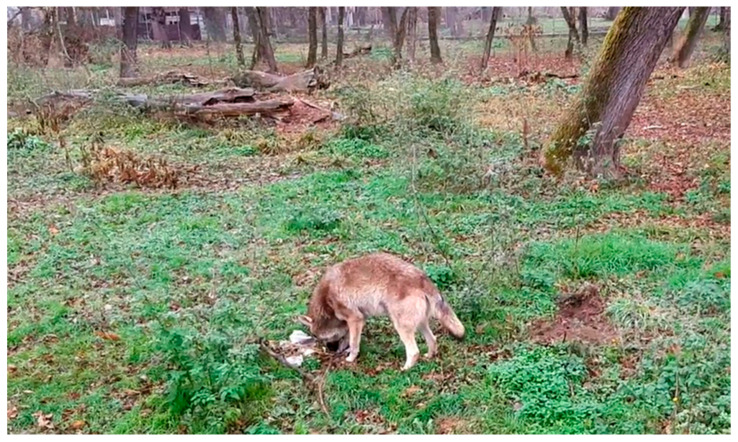
Adult female wolf sniffs deer/mouflon excrement at the Osijek Zoo in 2020. (Photo: Nikolina Boić).

**Figure 2:**
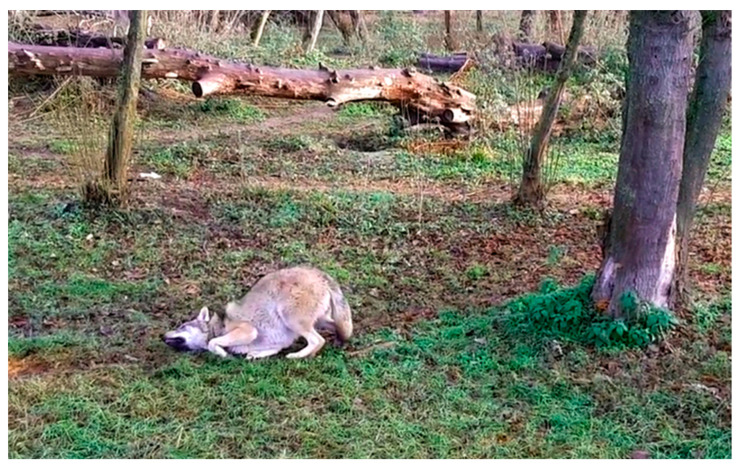
Osijek Zoo adult female wolf is rubbing her neck and shoulders in curry odour in 2020. (Photo: Nikolina Boić).

The expected behaviour of scent rolling was accompanied by the scattering and tearing of boxes and bags containing odours as well as urination on them. Scent rolling was triggered by the presence of a scented object and involved a sequence starting from the sniffing of the object (Figure 1) followed by the rubbing of the head against it, and then progressing to the rubbing of the neck, shoulders, and ultimately, the entire back against the scented object (Figure 2).

**Figure 3:**
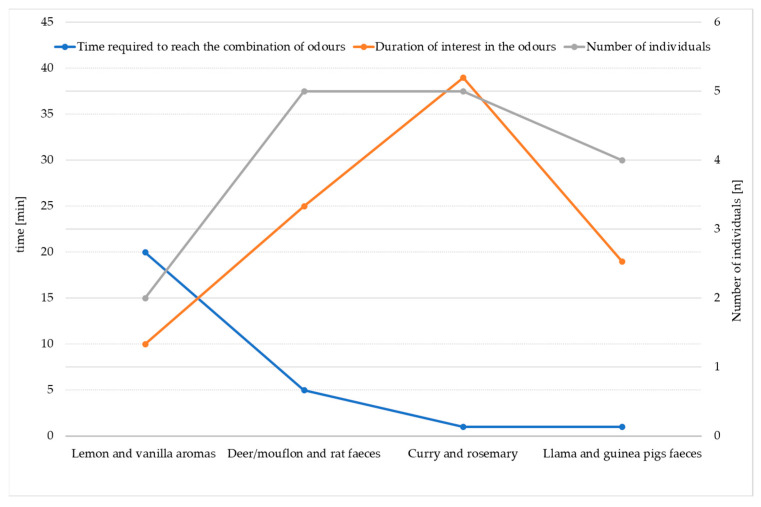
A parallel coordinate plot shows the time required to reach one of the two presented odours in minutes (total n = 27), the total duration of interest in minutes (total n = 93), shown by all five tested wolves and the number of captive wolves’ responses to odours (total n = 16) that showed interest in the two odours presented on the same day in 2020 (interest in the odour included various behaviour patterns detailed in Table 2).

In the initial phase of the study in November 2020, subjective observations indicated heightened caution and shyness among the wolves on the first day, particularly in their interactions with lemon and vanilla scents (Figure 3). This caution was manifested by prolonged hesitancy in approaching the scent sources, lowered head posture, and tentative approaches, suggesting a decreased exploration or interaction with the environment, or avoidance behaviours such as retreating or staying close to sheltered areas. Only two wolves, an adult female and one of the puppies, a male, exhibited interest in the aromas of vanilla and lemon, with a preference towards vanilla. The adult female took 20 min to approach the vanilla scent, followed immediately by the puppy. The female wolf showed a strong preference for the vanilla scent, actively participating in interest in it for about 10 min, while the puppy remained engaged for approximately 7 min. It is worth noting that the puppy simply followed its mother’s lead or possibly imitated her actions.

Subsequently, on the second day, all five wolves exhibited increased curiosity and reduced timidity, leading to a quicker response to the designated odours of deer/mouflon and rat faeces. This resulted in a total of 25 min of various interest in the scents among all individuals (Table 2, Figure 3). Throughout these observations, the male pup displayed several successive instances of scent rolling interspersed with periods of neutral, basic behaviour (i.e., walking or interacting with other individuals).

The highest level of interest in a completely new odour was particularly evident during exposure to the odours of curry and rosemary, with curry being the preferred scent for the wolves (Figure 3). These odours also aroused the longest duration of interest across all five individuals. Although the duration of scent rolling was not measured, subjective observations indicated a lesser intensity of scent-rolling behaviour. This indicates that through the period of 39 min, individuals frequently returned to the odours (predominantly curry bags), engaging primarily in sniffing, which is a precursor of complete scent-rolling behaviour. Although rosemary was the first to elicit interest in all individuals, that interest waned after 2 min. Consistently, the initial approach to each of these scents was made by the same two wolves—an adult female and a female pup. Subsequently, other pack members displayed interest, but with a greater level of caution compared with these two individuals. On the final day, the faeces of llamas and guinea pigs were inserted into the enclosure immediately after feeding. It was observed that these faeces did not arouse equally strong interest as the deer/mouflon and rat faeces. The preferred scent from the second day’s combination was deer/mouflon, while from the fourth day, it was guinea pig. The total duration of the observed responses exhibited by four wolves lasted 19 min, representing collective behaviour, with one individual (an adult male) showing no interest at all. 

In the second part of the study, carried out in October 2021, all four individuals were approaching the scented object immediately after its presentation (approximately 5 s). In most cases, they had already waited for the odour to be inserted. Wolves displayed a decrease in timidity compared with the preliminary experiment conducted a year earlier, as evidenced by their reduced latency in responding to odours. According to personal communication with the zoo caretakers, who observe and interact with the wolves daily, this reduced timidity is due to regular human interaction. This observation aligns with our study’s findings. However, it is intriguing that the total duration of interest in each odour was notably shorter in this instance (Table S2).

On both the first and last days of the experiment, when we monitored baseline and post-enrichment behaviours, we did not observe any behaviours typically associated with periods of scent enrichment. Instead, the wolves spent their time either resting or moving around the enclosure.

**Figure 4:**
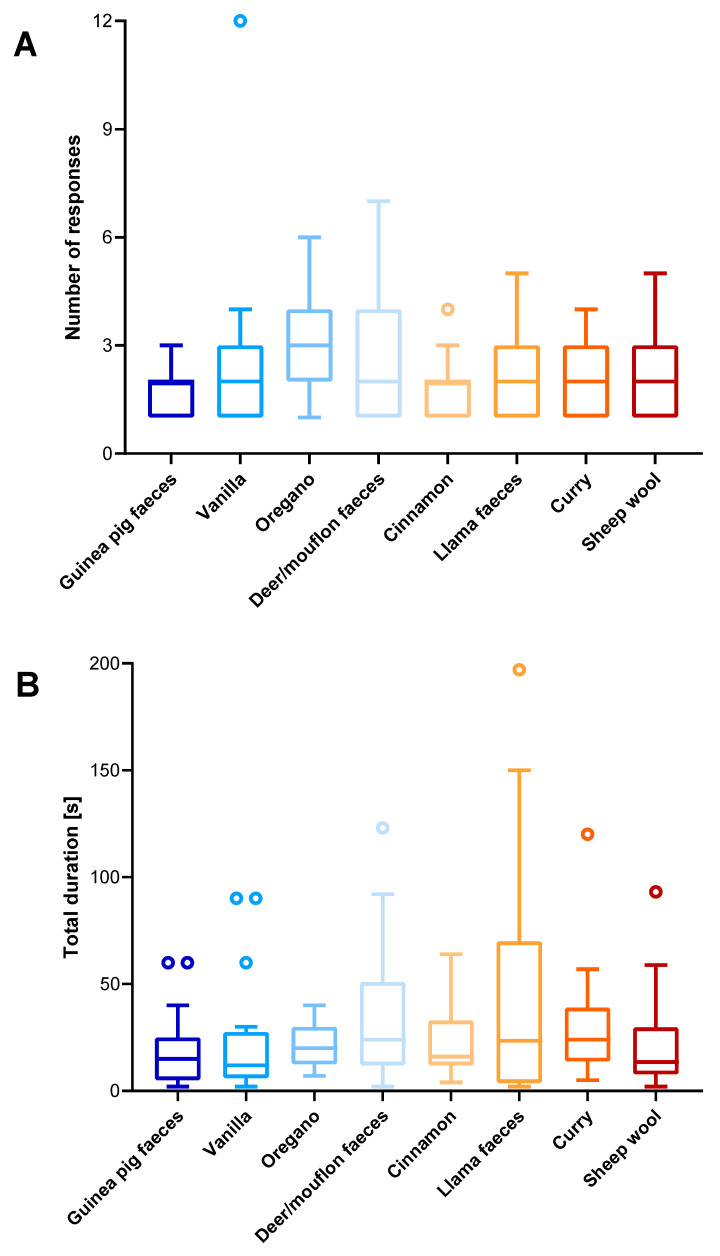
The numbers ((**A**); total n = 155) and the total durations of responses in seconds ((**B**); total n = 4352) of captive wolves (total n = 4) from the Osijek Zoo to various odours in 2021. Horizontal lines represent minimum and maximum values; each box represents two quartiles with the median as the central line. Outliers are represented by dots.

During days 2–8, the individuals devoted the majority of their time to the investigation of three kinds of faeces (guinea pig, deer/mouflon, and llama), with a share of 55% of the total duration of responses. Spices (vanilla, oregano, cinnamon, and curry) represented 34%, while sheep wool represented 11% of the total time captive wolves responded to various scents. The longest time spent responding to different odours was with llama faeces (1092 s, i.e., 18 min—9 responses), followed by deer/mouflon faeces (847 s, i.e., 14 min—66 responses), curry (538 s, i.e., 9 min—35 responses), sheep wool (474 s, i.e., 8 min—43 responses), guinea pig faeces (462 s, i.e., 8 min—44 responses), vanilla (404 s, i.e., 7 min—41 responses), cinnamon (338 s, i.e., 6 min—27 responses), and oregano (197 s, i.e., 3 min—27 responses), in that order (Figure 4, Tables S1 and S2). However, no statistically significant difference was found in the frequency and time spent responding to different odours used in the enrichment (*p* > 0.05 in both cases). Similarly, no statistically significant differences were found between the responses to animal-derived odours and the odours of spices and flavours used in this study.

**Figure 5:**
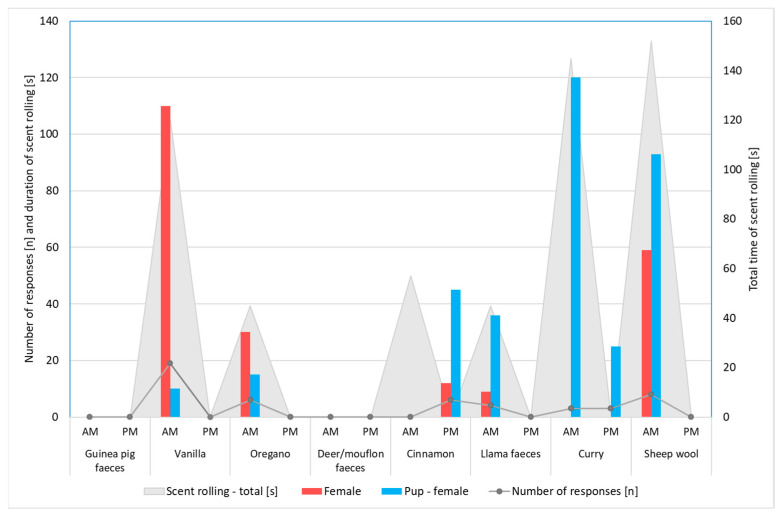
The number of responses to different odours (total n = 155), total time of scent rolling in seconds (total n = 564), and duration of scent rolling (in seconds; total n = 70.5) by individual wolves (total n = 4) from the Osijek Zoo. Only two individuals (both of them females) engaged in scent rolling behaviour.

In this second phase of the study, females were the first to approach the scents, followed by males, with no statistically significant differences observed in the frequency and duration of interest in scents between the sexes or individuals (*p* > 0.05 in both cases). Interestingly, scent-rolling behaviour was exclusively shown by females (alpha + pup), while this behaviour was not observed in males (Figure 5). Females engaged in scent rolling for an average of 70.5 s per day during the morning and afternoon sessions, totalling 12 scent rolling responses to scented objects over the course of all 8 days of olfactory enrichment (Table S3). Scent rolling did not occur in response to the scent of guinea pig faeces or deer/mouflon faeces but was observed during enrichment with all other odours. Based on our daily field observations, we noted that the scents presented tended to evoke greater interest in the morning (9:00–10:00) compared with the afternoon (16:00–17:00) (Figure 5). We expected that differences in responses to the same odour between the morning session and the afternoon one would be statistically supported; however, no statistical significance was found (*p* = 0.055). 

During the enrichment period, scent rolling lasted the longest when sheep’s wool was introduced (152 s) and the shortest during the oregano and llama excrement enrichment (45 s each) (these values correspond to the total duration of all instances of scent rolling shown by all tested wolves). When llama faeces were presented to the wolves, they mostly engaged in sniffing and tearing bags containing odours. On average, individuals spent the majority of their time per day tearing bags with scents (46 s, median = 25 s), while the least time was spent pawing at scented objects (3 s, median = 2.5 s), considering all objects (bags and boxes) and scents. 

**Figure 6:**
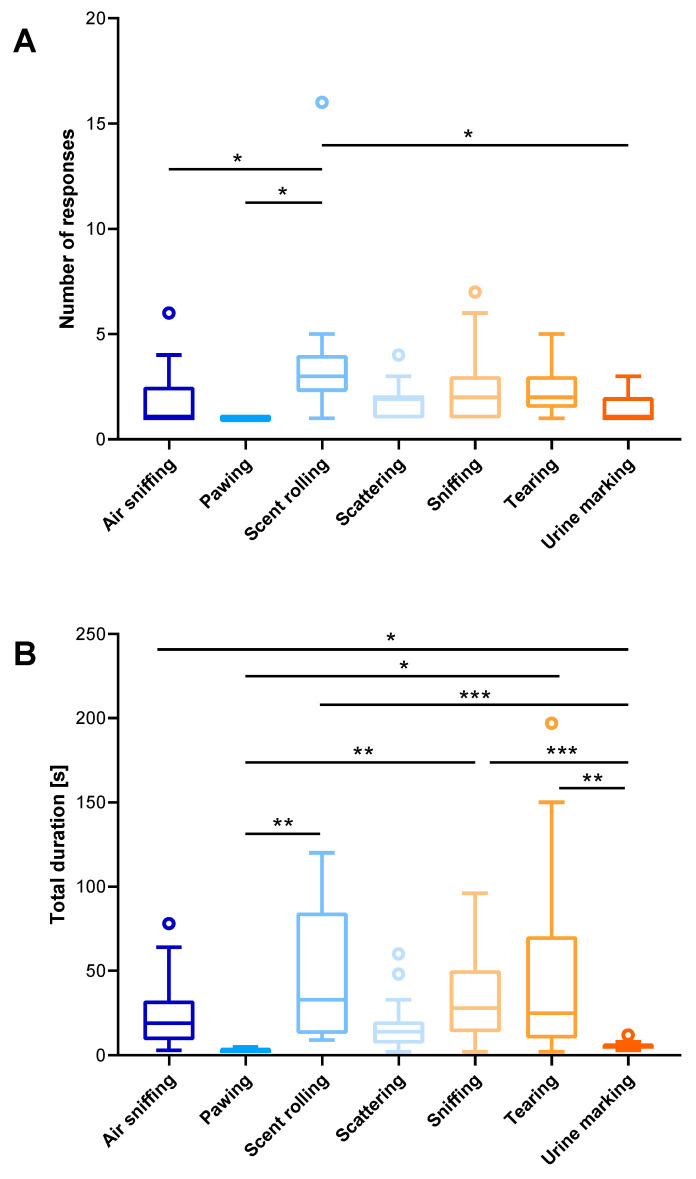
The number ((**A**); total n = 154) and total duration of responses in seconds ((**B**); total n = 4352) by captive wolves (total n = 4) at Osijek Zoo related to specific behaviours. Horizontal lines represent minimum and maximum values; each box represents two quartiles with the median as the central line. Outliers are represented by dots. Significant differences (Dunn’s post hoc) between the behaviours are noted with lines and asterisks: * (*p* < 0.05), ** (*p* < 0.01), *** (*p* < 0.001).

The numbers of responses and durations of specific behaviour patterns are shown in Figure 6; Tables S3 and S4. Significant differences in responses to scented objects were observed, particularly in scent rolling, which occurred significantly more frequently compared with other behaviours such as air sniffing (*p* = 0.008), pawing (*p* = 0.010), scattering (*p* = 0.033), and urine marking (*p* = 0.007). Regarding time spent performing each behaviour, the durations of urine marking were significantly lower than those of tearing (*p* = 0.0028), sniffing (*p* = 0.0001), scent rolling (*p* = 0.0006) and air sniffing (*p* = 0.0138). Time spent pawing was significantly lower in comparison with the time devoted to scent rolling (*p* = 0.0075), sniffing (*p* = 0.0094), and tearing (*p* = 0.0295). The rest of the time wolves spent moving around the enclosure, interacting with each other (playing), resting, and feeding (that last activity was observed only during the afternoon sessions).

## 4. Discussion

Environmental enrichment positively affects the behaviour of animals in captivity [28]. Different scents stimulate the olfactory sense in canids, which leads to an increase in their activity level [16]. The enrichment of the environment with scents is carried out to encourage animals to move more and engage more in the exploration of the environment [24,29]. Placing Grant’s gazelle faeces in front of the African wild dog’s enclosure encouraged the dogs to engage in more activities and show more social behaviour [16]. Olfactory enrichment studies in other animal species also confirmed the success of that treatment in increasing general activity [30] and decreasing stereotypies [31]. The exact way in which the olfactory enrichment works is unknown, but it is considered to be successful due to neophilia (attraction to novel stimuli) [2,32]. On the other hand, despite their concomitant neophobia and object-related fear, European grey wolves were found to respond to novel objects by repeated approaches and exploratory behaviour that might have involved responses to specific odour(s) [33]. In the study of Murtagh et al. (2020) [12], dogs did not show preferences when choosing between lavender-scented and rabbit-scented toys, which suggests that, in dogs, novelty still takes precedence over the evolutionary significance of scents in contrast to findings obtained for non-canid species. This means that dogs are more interested in the novelty of scented toys rather than in the scent itself. To our knowledge, previous studies on wolf behaviour have documented instances where previously unknown odours elicited heightened interest and activity in wolves, often resulting in species-specific behaviours such as scent rolling [4,34]. Wolves from the Osijek Zoo were never before in contact with any of the scents used in this study (except llama faeces), so neophilia could explain their interest in these odours. Although wolves might have had indirect exposure to some of the odours tested, it is important to note that the wolves were located at the edge of the zoo, quite distant from the animals whose faeces were used in the experiment. Therefore, the likelihood of prior exposure to the specific odours tested in the experiment is minimal. 

This study has shown that the smell of the herbivore faeces aroused the greatest interest among wolves in terms of response duration, with very weakly expressed scent rolling observed (except for llama faeces) in the first year of this study, and a complete absence of scent rolling in the second year. This result might have been obtained because wolves from Osijek Zoo were familiar with llama faeces from previous enrichment experiences (as part of the enrichment program at the Osijek Zoo). Also, according to Ryon et al. [4], herbivorous faeces did not induce wolves to engage in scent-rolling behaviour. The same authors observed that industrial odours such as perfumes and motor oil provoked the strongest reaction of scent rubbing in wolves, followed by the odours of faeces of other carnivorous species (pumas and bears), while food odours caused the weakest reaction and induced the least interest in the scent in question [4]. Environmental enrichment with sheep’s wool and curry scent prompted the most scent rolling behaviour in captive wolves from the Osijek Zoo. A study by John et al. (2019) [35] demonstrated that introducing sheep’s wool and curry scent into the wolves’ environment led to a significant increase in scent-rolling behaviour. This suggests that these particular scents triggered a strong olfactory response in wolves, prompting them to engage in scent-marking behaviours associated with territoriality and social communication. Goodman [36] compiled a list of odours that stimulated scent rolling in wolves, including the smell of urine and faeces of different animals, ash, various insect and dog repellents, fruits, human food like salted pork and tuna oil, and many others.

Wolves roll in marks left by other individuals of the same species but not in marks left by themselves [37]. This behaviour in canids plays a role in collecting other individuals’ odours, concealing their smells, or informing other members of the pack about the environment in which they are located [37,38]. Grey foxes, for example, rub their cheeks against scent marks left by pumas to accumulate puma scent. Foxes have been found to engage in that behaviour to cover their odour with the smell of pumas and thus deter other predators [24]. Wolves engage in rubbing mostly in response to uncommon odours [4]. Scent rolling can serve as a behaviour that would mask one’s scent or as camouflage [39,40], as is the case with grey foxes. Nevertheless, there is a theory that scent rolling plays a part in transmitting information from the environment to other individuals in the pack [8]. For example, if an individual encounters a carcass that would fulfil the nutritional needs of a pack, he will scent roll on the carcass to transmit the odour to the rest of the pack and thus inform them of a potential food source [41]. The role of odours in the information transmission and social interactions of wolves is poorly studied, yet communication by scents can be extremely effective considering their longevity in the environment [42]. Scent rolling has also been observed in other animals. The places on the body that are most often rubbed differ between different families. In hyenas, the rubbing of the neck, shoulders, and back has been observed, while cats prefer to rub parts of the head, especially the cheeks [3]. Various animals differ not only with respect to the parts of the body engaged in rubbing against the scents, but also with respect to the types of scents they prefer. Dogs, civets, and hyenas prefer the smells of food, excrement, and urine of herbivorous species, while cats (but also hyenas) very often choose the scent marks of other members of the same species [3]. 

The data on the contexts in which scent rolling occurs are still scarce. In cats and brown bears, scent rolling is known to be part of their scent-marking mechanism [2]. This behaviour in carnivorous species is often associated with social behaviour, especially during oestrus [43]. Scent rolling is a behaviour limited to terrestrial carnivores of a certain body size; that is, it has been observed only in large carnivores. Bertoni et al. (2023) [44] investigated the impact of introducing novel objects and scent stimuli on the behaviour of adult European wildcats at the Parco Natura Viva-Garda Zoological Park, Italy. Wildcats displayed altered behaviours, such as reduced individual exploration and increased inactivity, when exposed to F3-sprayed rags (F3: Facial Pheromone, which is naturally released by domestic cats when they rub their muzzle against objects or surfaces), suggesting the potential behavioural effects of semiochemicals. Exposure to F3-sprayed rags elicited altered behaviours in wildcats, including reduced individual exploration and increased inactivity, indicating potential behavioural effects of semiochemicals. This observation suggests that wildcats did not engage in scent rolling.

Scent rolling has never been observed in aquatic carnivores, which supports the theory that the role of this behaviour is to transfer odorous particles from the environment to the animal since water would wash out the odour from the hair of aquatic carnivores [3]. 

The limitations of the present research are mostly related to small sample size. Moreover, our small pack of captive wolves was housed in the same enclosure, which could result in a possible lack of independence of the data obtained for different animals as well as a lack of statistical significance in regards to the number and total duration of responses to various odours due to the high variability of the data (Figure 4). 

The research in the first year was conducted in the morning hours, while the research in the second year was extended to both morning and afternoon periods. During the morning, visitor numbers were fewer than in the afternoon. Although the number of visitors was lower in the morning, some visitors were, nevertheless, present, potentially causing distractions for the animals. These effects became particularly pronounced during the afternoon. In both wild habitats and zoos, the presence of human visitors and their interactions with wolves can influence wolf behaviour and stress levels, potentially leading to scent rolling as a response to changes in the environment, including the existence of unfamiliar scents or disturbances caused by human activities [45].

The natural fear or heightened caution that animals exhibit towards humans varies across species, with domestic cats and deer serving as examples. This comparison aims to highlight the range of fear responses among animals and how factors like habitat, familiarity, and past experiences with humans influence them. For example, wolves have shown higher alertness and more pronounced unusual behaviours in the presence of humans compared with some other animal species [46]. 

Another limitation of this study was the difficulty in tracking the duration of the animals’ interest in each scent due to scattering of the boxes and bags containing the scents by the tested animals. Additionally, in the afternoon sessions when scents were presented after feeding, the individuals were predominantly engaged in feeding and storing food, which might have reduced their attention to the odours. 

The most important novel aspects of the present research lie in the observation that the tested wolves engaged in scent rolling and displayed preferences for specific types of odours. In contrast to studies documenting the occurrence of scent-rolling behaviour in male wolves [6,46], during the second part of our study, only female wolves engaged in scent rolling. These results highlight a potentially gender-specific behaviour within wolf populations that has not been thoroughly documented before. Harrington and Asa (2003) [14] noted that female wolves, particularly those in breeding condition, may engage in scent-rolling behaviour as a way to communicate their reproductive status to other pack members. Overall, integrating the concepts of personality and behavioural syndromes into the studies of scent rolling in wolves can enhance our understanding of individual differences and the factors influencing this important territorial behaviour [5]. Some wolves may be more assertive, readily engaging in scent rolling, while others may be more timid, exhibiting less frequent or vigorous scent-rolling behaviour [6,47,48]. 

Moreover, this preliminary study fills a gap in the existing literature devoted to the behaviour of captive wolves displayed in response to different odours. By conducting this study, researchers have taken the first step in addressing this deficiency, providing valuable insights into the olfactory behaviours of wolves in zoo. This foundational research lays the groundwork for further exploration into the complexities of captive wolf behaviour and scent communication. Additionally, exploring a broader range of species would provide valuable insights into scent-rolling behavioural patterns and preferences across the Canidae family.

## 5. Conclusions

Scent rolling is a common and frequent behaviour observed in all large carnivores, including members of the canid family. Despite numerous theories regarding its purpose, this behaviour is influenced by the presence of some intense, and hitherto unknown, odours. Presumably, due to the lack of direct contact with members of other species, especially herbivores, the tested wolves found scents from this source particularly enticing, which could account for their heightened interest in such odours. Interestingly, during the second year of investigation, no scent rolling was observed in wolves exposed to the scent of certain herbivore faeces, such as guinea pig and deer/mouflon, while llama faeces, another type of herbivore faeces used during that period, did induce scent-rolling behaviour. Another noteworthy observation from this study was the predominant engagement of females in scent-rolling behaviour. Additionally, individual variations in behaviour and temperament may contribute to this observed gender gap. Such research holds significant importance for the welfare of animals kept in captivity, as it expands our knowledge of how to enrich their environments, mitigate the negative consequences of captivity, and ensure better living conditions. Considering the limitations of this study, particularly the small sample size of only two males and two females, it is acknowledged that further research is needed to thoroughly investigate the underlying mechanisms and implications of this potentially gender-specific behaviour. Expanding the sample size and including a more diverse range of individuals would enhance our understanding of wolf social dynamics and communication strategies.

## Figures and Tables

**Table 1:** A scheme of a 10-day trial with the wolf pack at the Osijek Zoo in 2021.

Day	Scent
1	No scent (observations of baseline behaviour)
2	Guinea pig faeces
3	Vanilla aroma
4	Oregano
5	Deer/mouflon faeces
6	Cinnamon
7	Llama faeces
8	Curry
9	Sheep’s wool
10	No scent (observations of post-enrichment behaviour

**Table 2:** An ethogram—category of sensory (olfactory) enrichment comprising behavioural patterns and their respective description, used in the study carried out in 2021 to investigate responses of four captive wolves to various scents.

Behavioural Patterns	Behaviour Description
Licking objects	Tongue contacts with the scented object
Sniffing	Lowering the head towards the scented object and sniffing it
Air sniffing	Raising the head in the direction of the scented object and sniffing the air
Urine marking	Marking the scented object with urine
Pawing	Touching the scented object with the paw/paws
Scattering	Taking the scented object in the mouth and scattering it in the enclosure
Tearing	Tearing off boxes/bags with scents
Scent rolling	Rubbing the head and body against the scented object

## Data Availability

Data will be made available upon request.

## Supplementary Materials

The following supporting information can be downloaded at: https://www.mdpi.com/article/10.3390/biology13060422/s1, Table S1. Descriptive statistics of the numbers of responses of captive wolves to various odours from 2021; Table S2. Descriptive statistics of time [s] spent by captive wolves responding to various odours; Table S3. The numbers of different behavioural responses of captive wolves (the data obtained for all 4 individuals pooled together) to various odours (the data for all 8 odours pooled); Table S4. Time [s] devoted by captive wolves to different behavioural responses to various odours.
