# Supplementary material for: Preliminary Study of Scent Rolling in Captive Wolves (Canis lupus L. 1758) in Response to Olfactory Enrichment
Source: Biology (Basel). 2024 Jun 6;13(6):422. doi: 10.3390/biology13060422 (PMC11200557; doi:10.3390/biology13060422)
Supplement: Supplementary file 1 [file biology-13-00422-s001.zip › biology-2973144-supplementary.pdf]

biology

MDPI

Supplementary information

Preliminary Study of Scent Rolling in Captive Wolves (*Canis lupus* L. 1758) in Response to Olfactory Enrichment

Nikolina Boić [1,2], Nikica Jukić [3], Alma Mikuška [1], Dora Bjedov [4,5], Mislav Kovačić [1], Tatjana Šalika-Todorović [3] and Mirta Sudarić Bogojević [1*]

[1] Department of Biology, Josip Juraj Strossmayer University of Osijek, Cara Hadrijana 8a, 31000 Osijek, Croatia
[2] Public institution Aquatika – Freshwater Aquarium Karlovac, Branka Čavlovića Čavleka 1a, 47000 Karlovac, Croatia
[3] UNIKOM d.o.o., Osijek Zoo, Sjevernodravska obala 1, 31000 Osijek, Croatia
[4] Centre for Ecology, Evolution and Environmental Changes (cE3c) & CHANGE – Global Change and Sustainability Institute, Faculdade de Ciências, Universidade de Lisboa, Campo Grande, 1749-016 Lisbon, Portugal
[5] Departamento de Biologia Animal, Faculdade de Ciências, Universidade de Lisboa, Campo Grande, 1749-016 Lisbon, Portugal
* Correspondence: mirta.sudaric@biologija.unios.hr

Table S1. Descriptive statistics of the numbers of responses of captive wolves to various odours from 2021.

	Guinea pig faeces	*Vanilla*	*Oregano*	*Deer/mouflon faeces*	*Cinnamon*	*Llama faeces*	*Curry*	*Sheep wool*
Number of responses	25	17	9	23	15	26	18	22
Minimum	1.000	1.000	1.000	1.000	1.000	1.000	1.000	1.000
25% Percentile	1.000	1.000	2.000	1.000	1.000	1.000	1.000	1.000
Median	2.000	2.000	3.000	2.000	2.000	2.000	2.000	2.000
75% Percentile	2.000	3.000	4.000	4.000	2.000	3.000	3.000	3.000
Maximum	3.000	12.00	6.000	7.000	4.000	5.000	4.000	5.000
Range	2.000	11.00	5.000	6.000	3.000	4.000	3.000	4.000
Mean	1.760	2.412	3.000	2.870	1.800	2.269	1.944	1.955
Std. Deviation	0.7234	2.647	1.500	1.866	0.9411	1.402	0.9984	1.133
Std. Error of Mean	0.1447	0.6420	0.5000	0.3891	0.2430	0.2749	0.2353	0.2415
Sum	44.00	41.00	27.00	66.00	27.00	59.00	35.00	43.00

Table S2. Descriptive statistics of time [s] spent by captive wolves responding to various odours.

	Guinea pig faeces	*Vanilla*	*Oregano*	*Deer/mouflon faeces*	*Cinnamon*	*Llama faeces*	*Curry*	*Sheep wool*
Number of responses	25	17	9	23	15	26	18	22
Minimum	2.000	2.000	7.000	2.000	4.000	2.000	5.000	2.000
25% Percentile	5.000	6.000	12.50	12.00	12.00	3.750	13.75	7.750
Median	15.00	12.00	20.00	24.00	16.00	23.50	24.00	13.50
75% Percentile	25.00	27.50	30.00	51.00	33.00	69.75	39.00	29.75
Maximum	60.00	90.00	40.00	123.0	64.00	197.0	120.0	93.00
Range	58.00	88.00	33.00	121.0	60.00	195.0	115.0	91.00
Mean	18.48	23.76	21.89	36.83	22.53	42.00	29.89	21.55
Std. Deviation	15.83	28.57	10.81	34.03	16.01	50.26	26.67	21.76
Std. Error of Mean	3.167	6.930	3.603	7.096	4.134	9.858	6.286	4.640
Sum	462.0	404.0	197.0	847.0	338.0	1092	538.0	474.0

Table S3. The numbers of different behavioural responses of captive wolves (the data obtained for all 4 individuals pooled together) to various odours (the data for all 8 odours pooled).

	Air sniffing	*Pawing*	*Scent rolling*	*Scattering*	*Sniffing*	*Tearing*	*Urine marking*
Number of responses	33	4	12	27	46	21	11
Minimum	1	1	1	1	1	1	1
25% Percentile	1	1	2.25	1	1	1.5	1
Median	1	1	3	2	2	2	1
75% Percentile	2.5	1	4	2	3	3	2
Maximum	6	1	16	4	7	5	3
Range	5	0	15	3	6	4	2
Mean	1.848	1	4.083	1.852	2.413	2.429	1.455
Std. Deviation	1.149	0	3.895	0.864	1.613	1.165	0.6876
Std. Error of Mean	0.2	0	1.125	0.1663	0.2379	0.2542	0.2073
Sum	61	4	49	50	111	51	16

Table S4. Time [s] devoted by captive wolves to different behavioural responses to various odours.

	Air sniffing	*Pawing*	*Scent rolling*	*Scattering*	*Sniffing*	*Tearing*	*Urine marking*
Number of responses	33	4	12	27	46	21	11
Minimum	3	2	9	2	2	2	3
25% Percentile	9	2	12.75	7	13.75	10	4
Median	19	2.5	33	14	28	25	5
75% Percentile	32.5	4.5	84.5	20	50.25	70.5	7
Maximum	78	5	120	60	96	197	12
Range	75	3	111	58	94	195	9
Mean	22.82	3	47	16.26	33.76	46.19	5.545
Std. Deviation	17.27	1.414	39.91	13.04	26.57	54.55	2.622
Std. Error of Mean	3.006	0.7071	11.52	2.51	3.918	11.9	0.7904
Sum	753	12	564	439	1553	970	61
